# Dr. Elmore Palmer

**Published:** 1909-12

**Authors:** 


Dr. Elmore Palmer, of Buffalo, died at his home, No. 309 Plymouth avenue, October 23, 1909. Dr. Palmer was born in Albion, Mich., in 1839, and took his medical degree at the University of Michigan. His wife and one daughter survive.

